# C1ql4 regulates breast cancer cell stemness and epithelial-mesenchymal transition through PI3K/AKT/NF-κB signaling pathway

**DOI:** 10.3389/fonc.2023.1192482

**Published:** 2023-05-31

**Authors:** Fan Xu, Jiali Wang, Shuman Zhen, Yuqing Duan, Qingshan Li, Lihua Liu

**Affiliations:** ^1^ Department of Tumor Immunotherapy, Fourth Hospital of Hebei Medical University, Shijiazhuang, China; ^2^ Departments of Oncology, Affiliated Hospital of Chengde Medical University, Chengde, China

**Keywords:** breast cancer, cancer stem cells, EMT, C1ql4, PI3K/Akt/NF-κB

## Abstract

**Background:**

The stemness characteristic of breast cancer (BC) is a crucial factor underlying cancer recurrence and metastasis after operative therapy and chemoradiotherapy. Understanding the potential mechanism of breast cancer stem cells (BCSCs) may ameliorate the prognosis of patients.

**Methods:**

We collected clinical specimens of BC patients for staining and statistical analysis to verify the expression status and clinical significance of complement C1q-like 4 (C1ql4). Western blot and qRT-PCR were employed to detect the expression of molecules. Flow cytometry was used to examine cell cycle, cell apoptosis and the portion of BCSCs. Wound healing and Transwell assays were used to detect cell metastasis. The effect of C1ql4 on breast cancer progression *in vivo* was examined in a nude mouse tumor bearing model.

**Results:**

Our clinical analysis showed that C1ql4 was highly expressed in BC tissues and cell lines, and the high expression of C1ql4 was significantly corelated with the malignancy of BC patients. Moreover, we also found that C1ql4 was overexpressed in BCSCs. C1ql4 knockdown suppressed the BCSC and EMT properties, promoted cell cycle progression, enhanced BC cell apoptosis, and inhibited cell migration and invasion, whereas the C1ql4 overexpression exhibited the opposite effects. Mechanistically, C1ql4 promoted the activation and nuclear location of NF-κB and the expression of downstream factors TNF-α and IL-1β. Moreover, inhibition of PI3K/AKT signaling suppressed the C1ql4-induced stemness and EMT.

**Conclusions:**

Our findings suggest that C1ql4 promotes the BC cell stemness and EMT *via* modulating the PI3K/AKT/NF-κB signaling, and provides a promising target for BC treatment.

## Introduction

Breast cancer (BC) is the most malignant and lethal cancer type in females (1, 2). Despite the development of early diagnosis and systematic therapeutic strategies, a significant number of BC patients still confront the poor prognosis caused by frequent recurrence and distant metastasis (3). Therefore, exploring the mechanism underlying the metastasis and invasion of BC is significant for identifying novel potential therapeutic targets and improving the prognosis of patients. There exists a particular portion of cells termed cancer stem cells (CSCs) in the tumor (4). These cells exhibit similar characteristics with stem cells, such as self-renewal and differentiation ability and positive expression of stemness-related genes, and are closely correlated with tumor progression, drug resistance, recurrence, and metastasis (5). Al-Hajj et al. first reported breast cancer stem cells (BCSCs) with specific markers (EpCAM/CD44+/CD24-/low) capable of initiating tumorigenesis *in vivo* (6). Epithelial-mesenchymal transition (EMT) is a biological process by which polar epithelial cells transform into transitional mesenchymal cells and acquire the ability to invade and migrate (7). It has been proven that CSCs show EMT features (8), and cancer cells can receive stem cell features *via* induction of EMT (9). Increasing studies have suggested that targeting BCSCs, and EMT are promising treatments for reversing recurrence in patients with BC (10).

C1ql (C1q-like) is the initiation molecule of the classical complement pathway, which can recognize immune complexes and initiate the classical pathways of the complement system. Four members of C1ql, namely C1ql1 (CTRP14), C1ql2 (CTRP10), C1ql3 (CTRP13), and C1ql4 (CTRP11), have been identified and are highly conserved during evolution (11, 12). Studies have reported that C1ql1 can act as a novel glioma promoter to promote the conversion of neural stem cell-like cells to glial cells from neurogenic cells, which is regarded as an early biomarker of malignant cells transformation (13). However, the role of C1ql4 in BC remains to be determined.

NF-κB is a crucial nuclear transcription factor involved in numerous biological processes such as apoptosis, immune response, inflammatory response, and metabolism by regulating the transcription and translation of various genes (14–16). Studies have revealed that the NF-κB signaling pathway is constitutively activated in multiple cancers, including BC, liver cancer, lung cancer, and melanoma, and plays a critical role in cancer progression (17, 18). It has been proven that excessive activation of SDF-1 can activate NF-κB signaling and induce BC cells EMT into BCSCs, indicating that the EMT process modulated by NF-κB pathway plays a significant role in the maintenance of BCSCs stemness and the recurrence and metastasis of BC (19). Therefore, proteins and compounds related to NF-κB activity have potential to be breakthroughs in targeted therapy for treating BC. Besides, a previous study reported that adiponectin, which contains a similar structure to C1ql4, promotes prostate cancer cell migration by activating NF-κB (20).

In this study, we determined the correlation of C1ql4 with BC stemness and EMT and discovered a potential promoting role of C1ql4 in BCSCs. Further detection of molecular mechanisms revealed that C1ql4 functions through PI3K/AKT signaling to activate the NF-κB function in BC cells. Our work provides a novel target to suppress cancer cell stemness and EMT for BC treatment.

## Materials and methods

### Clinical samples and immunohistochemistry

The breast cancer and para-carcinoma tissues were obtained from the Breast Surgery Department of Affiliated Hospital of Chengde Medical University, Chengde, China, form May 2015 to April 2017. None of the patients received any antitumor therapy before surgery. IHC staining was performed as described before (21). The antibodies were used as follows: C1ql4, β-actin (1:100 dilution, CST, USA).

### Cell lines

Breast cancer cell lines BT549, MDA-MB-231, MCF-7, and HCC1428 were bought from American Type Culture Collection (ATCC) and cultured in DMEM (Gibco, USA) with 10% fetal bovine serum (FBS; Gibco, USA) and 1% streptomycin/penicillin (Gibco, USA) in a humidified 37°C incubator that contains 5% CO_2_.

### Cell transfection

Cells were seeded into a 6-well plate and cultured at incubator overnight. The C1q14 overexpression vectors (OE-C1q14), siRNA targeting C1q14 (si-C1q14), and negative control (NC) were purchased from Genepharma (Shanghai, China). Cell transfection was conducted using Lipofectamine 2000 (Invitrogen, USA) according to the manufacturer’s introduction. After transfection for 72 h, cells were collected for the following studies.

### Cell treatment

The cycloheximide (200 ng/mL), bortezomib (0.6 nM), AKT inhibitor (AZD5363, 3 nM), IKK inhibitor (PS-1145, 88 nM), IκB inhibitor (BAY11-7082, 50 μM), and NF-κB inhibitor (JSH23, 7.1 μM) were used for the treatment of cells. Cells were transfected for 48 h, followed by treatment with corresponding inhibitors and activators for 24 h. Cells were then collected for the following studies. All inhibitors and activators were bought from MCE (USA) and used as per the manufacturer’s protocols.

### Cell cycle and apoptosis

Cell cycle and apoptosis were detected by conducting flow cytometry. For detecting cell cycle, cells were collected, fixed in ice alcohol, and stained with propidium iodide (PI) at 4°C for 30 min. The cells were stained with Annexin V and PI for 30 min for apoptosis detection and checked with flow cytometry.

### Transwell assay

Cells were seeded in serum-free medium, and placed into the upper chamber of Transwell that was covered with Matrigel (Corning, USA). The lower chamber was filled with a complete medium. After incubation for 24 h, the upper section was collected and stained with crystal violet. The invaded cells were captured and counted under a microscope (Leica, Germany).

### Wound healing assay

Cells were placed into a 6-well plate and incubated overnight to form monolayer, which was then scratched by sterile 10 µL pipette. The cells were then washed with PBS to remove debris and cultured in serum-free medium for 24 h. Images of wounds were captured at 0, 12, and 24 h.

### Sphere formation assay

After indicated treatment, cells were suspended in serum-free DMEM/F12 (HyClone, USA) that contains 20 ng/mL EGF (Promega, USA), 20 ng/mL b-FGF (Promega, USA), and 2% B27 (Invitrogen, USA) as single cells. The cells were then incubated in an ultra-low attachment 96-well plate (Corning, USA) at a density of 5×10^3^ cells per well. After incubation for 10 days, mammospheres were captured under a microscope (Leica, Germany).

### Detection of BCSCs

The cells were collected and suspended in PBS that contained anti-CD44 APC and anti-CD24 PE antibodies on ice for 30 min. The samples were then analyzed by flow cytometer (BD Bioscience, USA).

### Western blot assay

Cells and tissues were lysed in RIPA buffer (Beyotime, China) that contains phosphatase inhibitors (Sigma, USA). Cell lysates were separated in 8% to 12% SDS-PAGE gel and transferred onto polyvinylidene fluoride membrane (Millipore, USA). After blocking in 5% non-fat milk for 2 h, the protein bands were probed with specific primary antibodies at 4 ˚C overnight. The next day, the protein bands were hatched with horseradish peroxidase-conjugated secondary antibody for 1 h at room temperature. After incubation with ECL solution (Millipore, Germany), the probed protein bands were visualized on an imaging system. The primary antibodies and secondary antibodies were brought from Abcam (USA).

### Quantitative real time PCR assay

Total RNAs were extracted from cells and tissues using Trizol reagent (Invitrogen, USA). A total of 1 μg mRNA was reverse-transcribed into cDNA. RNA expression level was analyzed by real-time fluorescent quantitative PCR using SYBR Green/ROX qPCR Master Mix (Qiagen, USA). The relative expression level of target gene was measured by 2^-ΔΔCt^ method. β-actin was used as an internal control. The primer sequences were listed as following:

**Table:** 

Gene	Sense primer	Antisense primer
C1ql4	5'-AGTACAGCACCT TCTCCGGCTTC-3'	5'-CCGCCAGGCTCTCAAAGGGT-3'
CD44	5'-CTGCCGCTTTG CAGGTGTA-3'	5'-CATTGTGGGCAAGGTGCTATT-3'
Nanog	5'-TCCCGAGAAAAG ATTAGTCAGCA-3'	5'-AGTGGGGCACCTGTTTAACTT-3'
OCT4	5'-CCGAAAGAGAAA GCGAACCAGT-3'	5'-ACATCCTTCTCGAGCCCAAG-3'
E-Cadherin	5'-CGAGAGCTACA CGTTCACGG-3'	5'-GGGTGTCGAGGGAAAAATAGG-3'
N-Cadherin	5'-TTTGATGGAGGTC TCCTAACACC-3'	5'-ACGTTTAACACGTTGGAAATGTG-3'
Vimentin	5'-GCCCTAGACGA ACTGGGTC-3'	5'-GGCTGCAACTGCCTAATGAG-3'
TNFα	5'-CCTCTCTCTAATC AGCCCTCTG-3'	5'-GAGGACCTGGGAGTAGATGAG-3'
IL-1β	5'-CTCTCTCCTTTC AGGGCCAA-3'	5'-GCGGTTGCTCATCAGAATGT-3'
β-actin	5'-CTCCTGAGCGCAA GTACTCT-3'	5'-TACTCCTGCTTGCTGATCCAC-3'

### Immunofluorescence assay

Cells were placed in confocal dishes (Nest, USA) for 24 h and fixed in 4% paraformaldehyde (PFA). After permeabilization by 0.1% Triton X-100 (Beyotime, China). The cells were blocked with sheep serum at room temperature for 30 min and then stained with the anti-NF-κB primary antibody (Abcam, USA) overnight at 4°C. The samples were then incubated with the fluorescence labelled secondary antibody for 45 min at room temperature. The nuclei were stained with DAPI for 10 min. Images were taken under confocal laser scanning fluorescence (Leica, Germany).

### Mouse xenograft model

Female NOD/SCID nude mice aged 6-8 weeks old were bought from Vital River Laboratory (Beijing, China). Cancer cells with luciferase tag (MCF-7-luc and MDA-MB-231-luc) were used for *in vivo* study. C1q14 overexpression vectors or sh-C1q14 vectors were encapsulated with lentivirus and used to infect MCF-7-luc cells or MDA-MB-231-luc cells, respectively. After that, a total of 1×10^6^ cells in 200 µL PBS were injected into each mouse through tail vein. The cell distribution was observed using *in vivo* imaging system (IVIS) after cell injection. The lung tissues were checked by HE staining and detected the portion of BCSCs and EMT.

### Statistics

All data in this work were shown as the mean ± standard deviation (SD) and analyzed by GraphPad Prism and SPSS 20.0 software. The independent t-test and one-way analysis of variance were applied for comparison between two groups or multiple groups. *p* < 0.05 was considered as statistically significant difference.

## Results

### C1q14 overexpression was remarkably correlated with poor prognosis of BC patients

**Figure 1:**
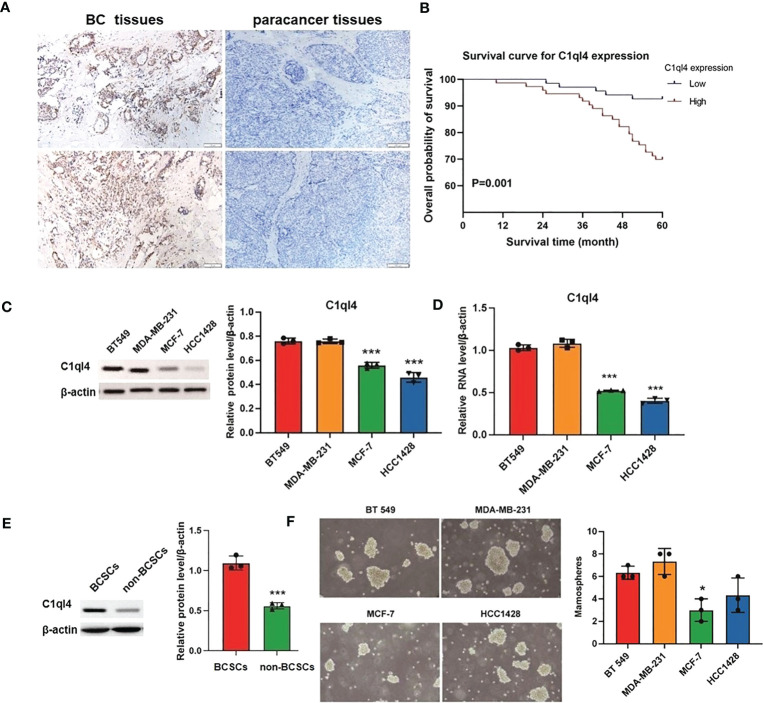
C1ql4 was significantly associated with poor prognosis and stemness of BC. **(A)** IHC for C1ql4 expression status in BC tissues (n = 141) and para-cancer tissues (n = 141). **(B)** Kaplan-Meier analysis of the relationship between C1ql4 expression status with OS indicated that C1ql4 expression was negatively correlated with OS. **(C)** Western blot for C1ql4 expression status in BC cell lines. **(D)** qPCR for C1ql4 expression levels in BC cell lines. **(E)** Western blot for C1ql4 expression status in BCSCs and non-BCSCs. **(F)** Sphere-formation experiments for differential C1ql4 expression in BC cell lines. *p < 0.05, ***p < 0.001.

To get an overview of the expression signature of C1ql4 in BC tissues and its relationship with the clinicopathological parameters of the BC patients, we employed IHC assay. As shown in **Figure 1A**, the expression of C1ql4 was significantly higher in BC tissues, compared to para-carcinoma tissues. Besides, IHC also showed that the C1ql4 was mainly expressed in the cytoplasm of BC cells. Importantly, high C1ql4 expression was obviously associated with tumor size, lymph node metastasis, TNM stage, PR expression status and molecular typing, but not with age, tumor differentiation, HER-2 expression status, ER expression status and Ki-67 expression status (**Table 1**). We obtained the correlation between the prognosis of BC patients and the expression of C1ql4 by performing Kaplan-Meier analysis. As was shown in **Figure 1B**, there was a significant relationship between the expression of C1ql4 and overall survival (OS) in BC patients.

**Table 1:** The relationship between C1ql4 expression and clinicopathologic features in BC.

Features	n	C1ql4 expression status [n(%)]		χ2	*P-*Value
		high (n=73)	low (n=68)		
Age				1.087	0.401
<40 years	14	9(64.3)	5(35.7)		
≥40 years	127	64(50.4)	63(49.6)		
Tumor size				5.909	0.018^*^
≤2 cm	61	24(39.3)	37(60.7)		
>2cm	80	48(60.0)	32(40.0)		
Lymph node metastasis				9.659	0.002^**^
negative	67	25(37.3)	42(62.7)		
positive	74	47(63.5)	27(36.5)		
Pathological grade				0.910	0.393
I	13	5(38.5)	8(61.5)		
II~III	128	67(52.3)	61(47.7)		
TNM stage				13.274	0.001^***^
I~II	103	43(41.7)	60(58.3)		
III~IV	38	29(76.3)	9(23.7)		
ER				4.474	0.051
negative	49	31(63.3)	18(36.7)		
positive	92	41(44.6)	51(55.4)		
PR				4.022	0.06
negative	59	36(61.0)	23(39.0)		
positive	82	36(43.9)	46(56.1)		
Her-2				1.708	0.247
negative	105	57(54.3)	48(45.7)		
positive	36	15(41.7)	21(58.3)		
Ki-67				0.291	0.689
≤14%	32	15(46.9)	17(53.1)		
>14%	109	57(52.3)	52(47.7)		
Molecular subtype				8.985	0.003^**^
Non-triple negative	112	50(44.6)	62(55.4)		
Triple negative	29	22(75.9)	7(24.1)		

*P<0.05, **P<0.001, ***P<0.001 vs C1ql4 low expression group.

Furthermore, we evaluated the expression status of C1ql4 in several BC cell lines. The results from western blotting assay and qPCR assay showed that the level of C1q14 was significantly higher in BT 549 and MDA-MB-231 cells, compared with the MCF-7 and HCC1428 cells (**Figures 1C, D**). These data indicated that C1ql4 was highly expressed in BC, and the expression of C1ql4 was a risk factor for poor prognosis of BC patients.

### C1ql4 is highly expressed in BCSC

To determine the expression status of the C1ql4 gene in breast cancer stem cells, we isolated CD44+CD24- cell populations from MCF-7 cell lines by flow cytometry. We found that C1ql4 was highly expressed in CD44+CD24- cells compared to their counterpart CD24+ or CD24-CD44- cells in regards to protein levels (**Figure 1E**). Next, we used BC cell lines to perform sphere-formation experiments and measured the expression of the C1ql4. The results showed that C1ql4 was significantly elevated in spherical cells compared to their counterpart adherent cells (**Figure 1F**). Taken together, these results suggested that elevated expression of C1ql4 was enriched in BCSCs and was a novel feature of BCSC-like subpopulation.

### C1q14 is crucial for the maintenance of BC cell stemness

**Figure 2:**
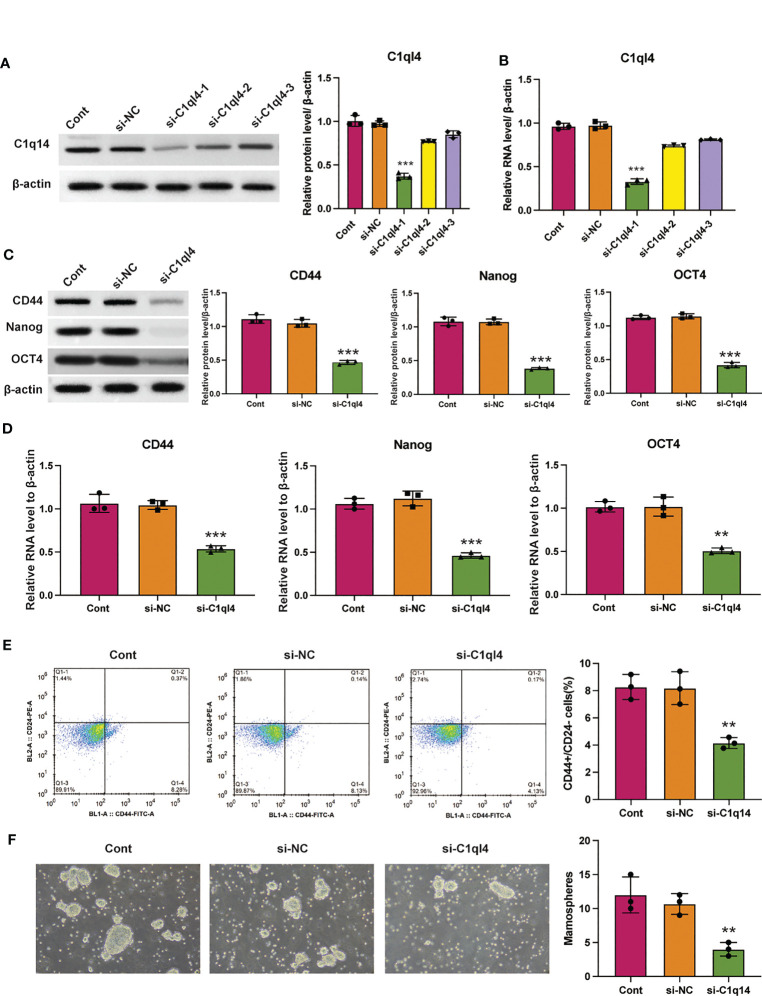
Knockdown of C1q14 suppresses breast cancer cell stemness. MDA-MB-231 cells were transfected with C1q14 siRNAs. The **(A)** protein and **(B)** RNA levels of C1q14 were measured by western blotting and qPCR assay, and the results showed that C1ql4 was attenuated in si-C1ql4 group. The **(C)** protein and **(D)** RNA levels of CD44, OCT4, and Nanog in different group cells were measured by western blotting and qPCR assay, and the result indicated that C1ql4 knockdown could inhibit the expression of CD44, OCT4, and Nanog. **(E)** The portion of CD44+CD24-/low cells in different group cells were measured by flow cytometry. **(F)** The effect of C1q14 knockdown on sphere formation ability in MDA-MB-231 cells. **p < 0.01, ***p < 0.001.

**Figure 3:**
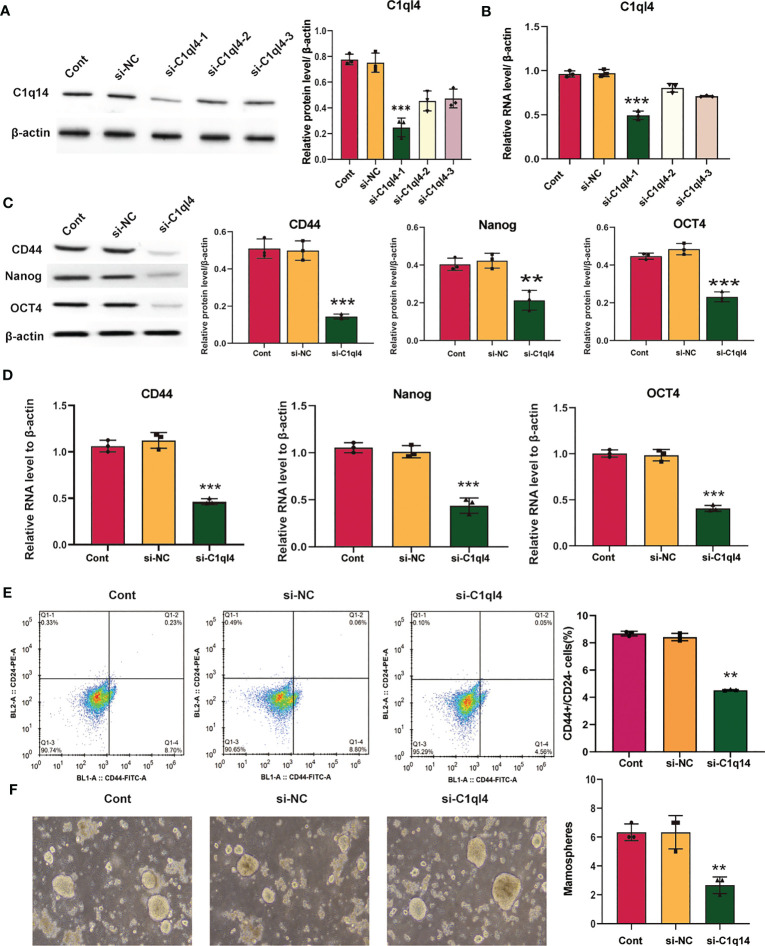
Knockdown of C1q14 suppresses the stemness of breast cancer BT549 cells. BT549 cells were transfected with C1q14 siRNAs. The **(A)** protein and **(B)** RNA levels of C1q14 were measured by western blotting and qPCR assay, and the results showed that C1ql4 was attenuated in si-C1ql4 group. The **(C)** protein and **(D)** RNA levels of CD44, OCT4, and Nanog in different group cells were measured by western blotting and qPCR assay, and the result indicated that C1ql4 knockdown could inhibit the expression of CD44, OCT4, and Nanog. **(E)** The portion of CD44+CD24-/low cells in different group cells were measured by flow cytometry. **(F)** The effect of C1q14 knockdown on sphere formation ability in BT549 cells. **p < 0.01, ***p < 0.001.

**Figure 4:**
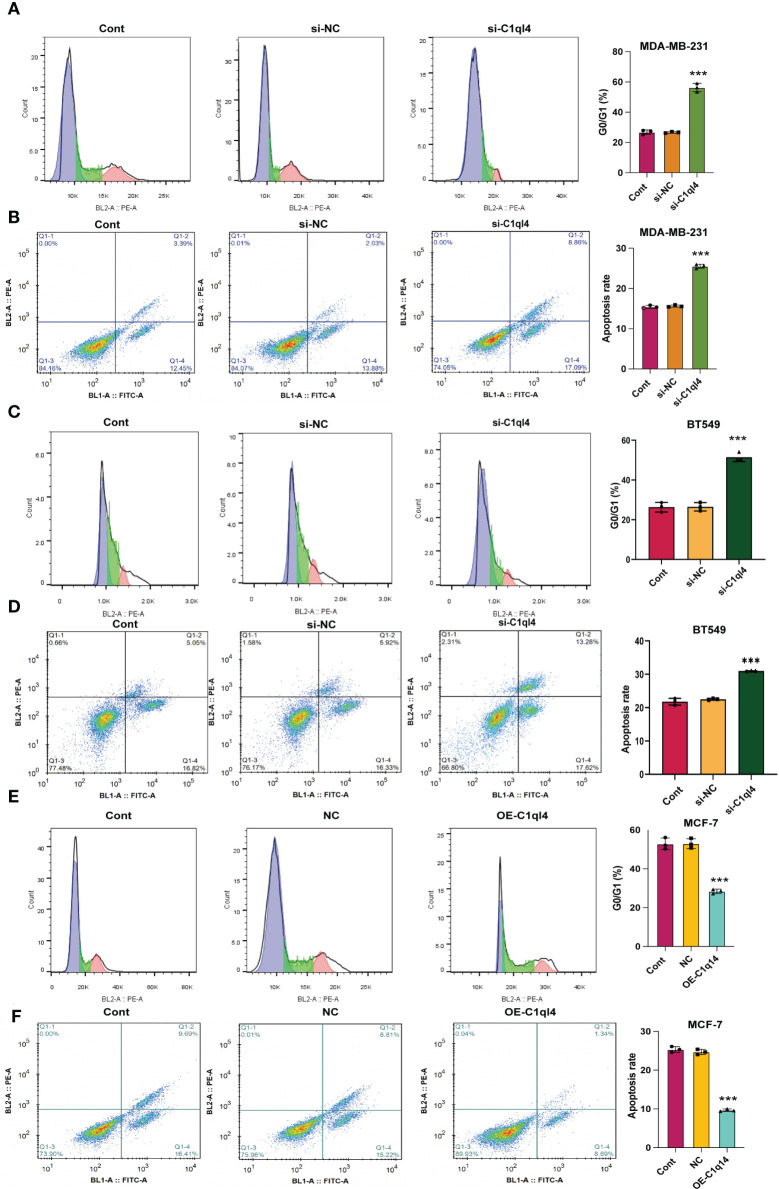
C1q14 knockdown affects proliferation and survival of BC cells. The effect of C1ql4 knockdown on the **(A, C)** cell cycle and **(B, D)** cell apoptosis was measured by flow cytometry, and the results suggested that C1ql4 knockdown could facilitate cell cycle arrest and apoptosis of MDA-MB-231 and BT549 cells. The effect of C1ql4 overexpression on the **(E)** cell cycle and **(F)** cell apoptosis was measured by flow cytometry, and the results showed that C1ql4 overexpression could inhibit cell cycle arrest and apoptosis of MCF-7 cells. ***p < 0.001.

Based on the result that C1ql4 was upregulated in BCSCs, we further explored whether C1ql4 functioned in BCSCs behavior. To achieve this goal, we knocked down C1ql4 in MDA-MB-231 and BT549 cells by using three different siRNAs, qPCR and Western blot assays showed that C1ql4 expression could be obviously decreased by siRNA#1 in MDA-MB-231 cells (**Figures 2A, B**) and BT549 cells (**Figures 3A, B**). Then, we observed that the expression of BCSCs markers, CD44, OCT4, and Nanog were significantly attenuated in C1ql4 knockdown MDA-MB-231 cells (**Figures 2C, D**) and BT549 cells (**Figures 3C, D**) compared with their counterpart cells, respectively. Additionally, C1ql4 knockdown notably decreased the portion of CD44+CD24-/low cells in MDA-MB-231 cells (**Figure 2E**) and BT549 cells (**Figure 3E**). Furthermore, compared to the control cells, C1ql4 knockdown obviously suppress the formation of spheroids in MDA-MB-231 cells (**Figure 2F**) and BT549 cells (**Figure 3F**). Subsequently, we determined the effects of C1ql4 in proliferation and apoptosis. C1ql4 knockdown could facilitate the cell cycle arrest of MDA-MB-231 cells (**Figure 4A**) and BT549 cells (**Figure 4C**) at G0/G1 phase. Moreover, the apoptotic cells of C1ql4 lost MDA-MB-231 and BT549 cells were significantly higher comparing the control cells (**Figures 4B, D**).

**Figure 5:**
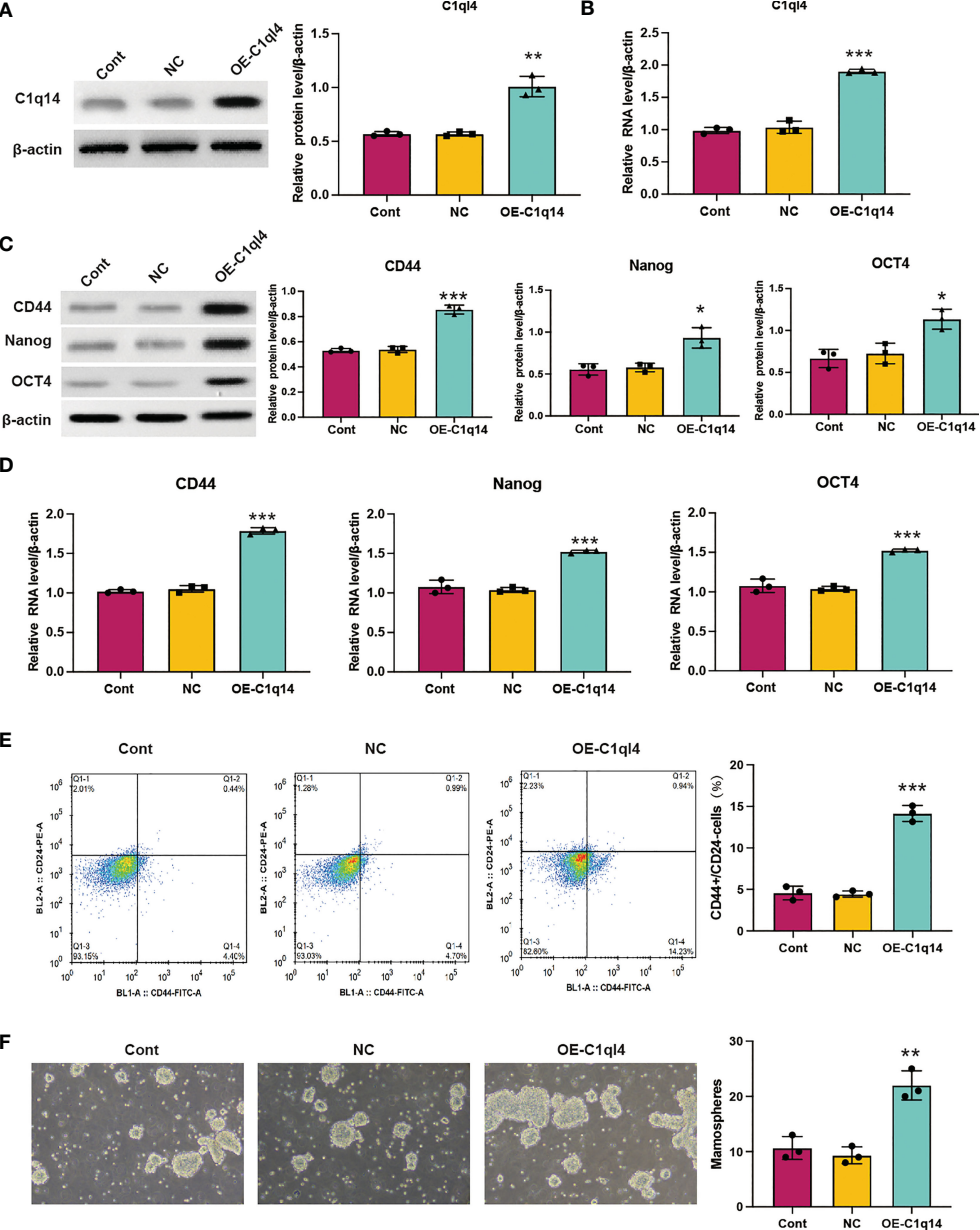
Overexpression of C1q14 boosts breast cancer cell stemness. MCF-7 cells were transfected with C1q14 overexpression vectors. The **(A)** protein and **(B)** RNA levels of C1q14 were measured by western blotting and qPCR assay. The **(C)** protein and **(D)** RNA levels of CD44, OCT4, and Nanog in different group cells were measured by western blotting and qPCR assay, and the results demonstrated that C1ql4 overexpression could increase the expression of CD44, OCT4, and Nanog in MCF-7 cells. **(E)** The portion of CD44+CD24-/low cells in different group cells were measured by flow cytometry. **(F)** The effect of C1q14 overexpression on sphere formation ability in MCF-7 cells. *p < 0.05, **p < 0.01, ***p < 0.001.

In order to clarify the function of C1ql4 in BCSCs behavior more rigorously, we overexpressed C1ql4 in MCF-7 cells using a lentivirus transduction system (**Figures 5A, B**). In contrast to C1ql4 knockdown, C1ql4 overexpression remarkably increased the expression levels of BCSC markers, CD44, OCT4, and Nanog (**Figures 5C, D**). C1q14 overexpression also increased the portion of CD44+CD24-/low cells in MCF-7 cells (**Figure 5E**), as well as enhanced the sphere formation ability (**Figure 5F**). Then, overexpression of C1q14 significantly suppressed the cell cycle arrest and apoptosis of the MCF-7 cells (**Figures 4E, F**). These data demonstrated that C1ql4 could positively regulate the stemness characteristics of BC cells.

### C1q14 modulates metastasis of BC cell *via* facilitating EMT

**Figure 6:**
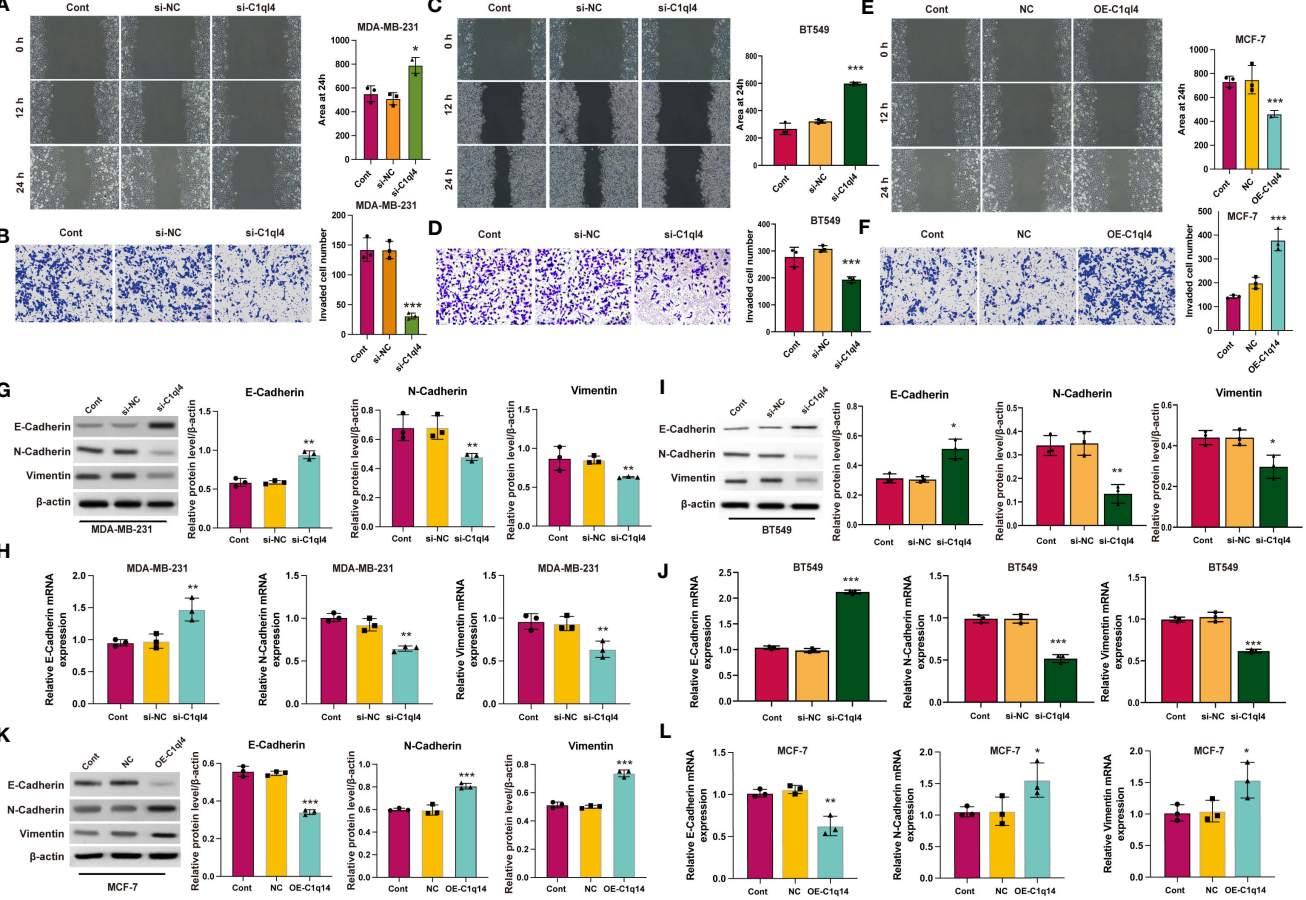
C1q14 modulates metastasis *via* affecting EMT. **(A, B)** The effect of C1ql4 knockdown on the migration and invasion of MDA-MB-231 cells. (**C, D**) The effect of C1ql4 knockdown on the migration and invasion of BT549 cells. **(E, F)** The effect of C1ql4 overexpression on the migration and invasion of MCF-7 cells. The (**G, I**) protein and (**H, J**) RNA levels of E-cadherin, N-cadherin and Vimentin in C1ql4 knockdown MDA-MB-231 and BT549 cells were measured by western blotting and qPCR assay. The **(K)** protein and **(L)** RNA levels of E-cadherin, N-cadherin and Vimentin in C1ql4 overexpression MCF-7 cells were measured by western blotting and qPCR assay. *p < 0.05, **p < 0.01, ***p < 0.001.

Currently, the underlying biological mechanism accounting for the elevated expression of C1ql4 in BC remains unclear. To demonstrate the function of C1ql4 in BC progression, we employed would healing and Transwell experiments to clarify the role of C1ql4 in invasion and migration in BC cells. Knockdown of C1q14 in MDA-MB-231 and BT549 cells suppressed the wound healing (**Figures 6A, C**) and cell invasion (**Figures 6B, D**). The overexpression of C1q14 increased the wound healing speed of MCF-7 cells (**Figure 6E**), simultaneously elevated the invasion of MCF-7 cells through Transwell chambers (**Figure 6F**).

EMT is a dynamic process in which epithelial cells acquire enhanced mobility and invasive properties by losing cell-cell adhesion structures and polarity. To further detect the biological mechanism of C1ql4 on cell migration and invasion, we focused on the identification whether C1ql4 affected EMT. We detected typical EMT markers (E-cadherin, N-cadherin, Vimentin) by western blotting. As shown in **Figure 6**, the protein (**Figures 6G, I**) and RNA (**Figures 6H, J**) levels of epithelial biomarker E-cadherin was lower and the mesenchymal biomarkers, N-cadherin and Vimentin were higher in C1ql4-knockdown MDA-MB-231 and BT549 cells. Overexpression of C1q14 notably repressed the expression of E-cadherin and upregulated the levels of N-cadherin and Vimentin (**Figures 6K, L**) in MCF-7 cells, suggesting the enhanced EMT. These results suggested that C1ql4 was involved in modulating EMT in breast cancer progression and facilitates the invasion and migration capacity of BC cells.

### C1q14 drives cancer stemness *via* PI3K/AKT/NF-κB signaling pathway in BC cells

**Figure 7:**
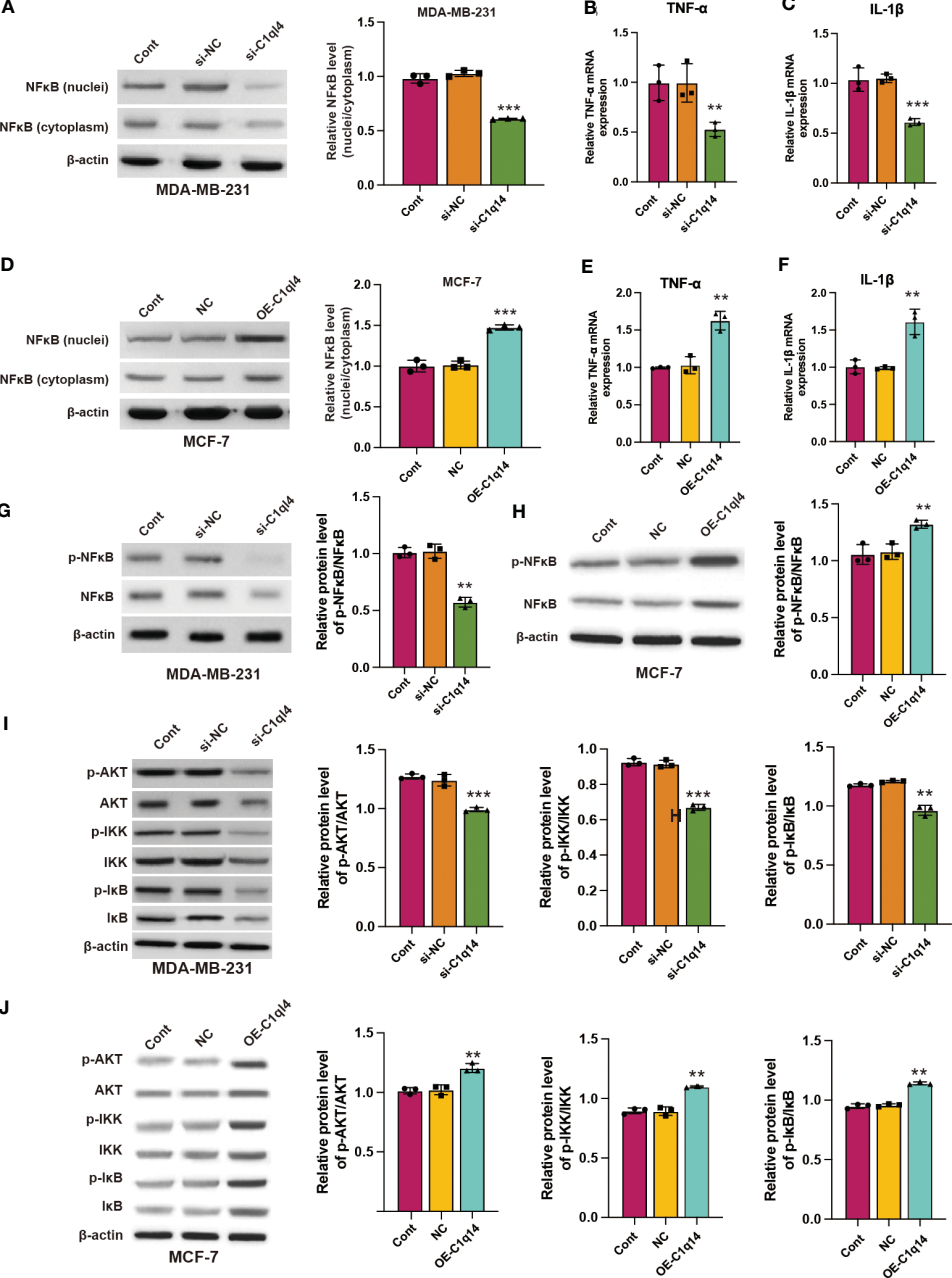
C1q14 regulates the activation of PI3K/AKT/NF-κB signaling pathway in breast cancer cells. **(A)** The protein level of NF-κB in nuclei and cytoplasm of MDA-MB-231 cells were checked by western blotting. **(B, C)** The RNA levels of TNF-α and IL-1β in MDA-MB-231 cells were measured by qPCR assay. Then the **(D)** protein level of NF-κB in nuclei and cytoplasm of MCF-7 was checked by western blotting. **(E, F)** The RNA levels of TNF-α and IL-1β in MCF-7 cells were measured by qPCR assay. **(G, H)** The protein levels of p-NF-κB and NF-κB in **(G)** MDA-MB-231 and **(H)** MCF-7 cells were measured by western blotting. **(I, J)** The protein levels of total and phosphorylated AKT, NF-κB, IKK, and IκB in **(I)** MDA-MB-231 and **(J)** MCF-7 cells were measured by western blotting. **p < 0.01, ***p < 0.001.

We observed that C1q14 knockdown decreased the nuclei to cytoplasm ratio of NF-κB location in MDA-MB-231 cells (**Figure 7A**), simultaneously inhibited the RNA levels of NF-κB target TNF-α and IL-1β (**Figures 7B, C**). In contrast, overexpression of C1q14 increased the location of NF-κB in MCF-7 cell nuclei (**Figure 7D**) and upregulated the expression of TNF-α and IL-1β (**Figures 7E, F**). Besides, knockdown of C1q14 suppressed the phosphorylation of NF-κB (**Figure 7G**), whereas overexpression of C1q14 increased the level of phosphorylated NF-κB (**Figure 7H**). Noteworthy, knockdown and overexpression of C1q14 suppressed and enhanced the level of phosphorylated AKT, IKK, and IκB (**Figures 7I, J**), respectively.

**Figure 8:**
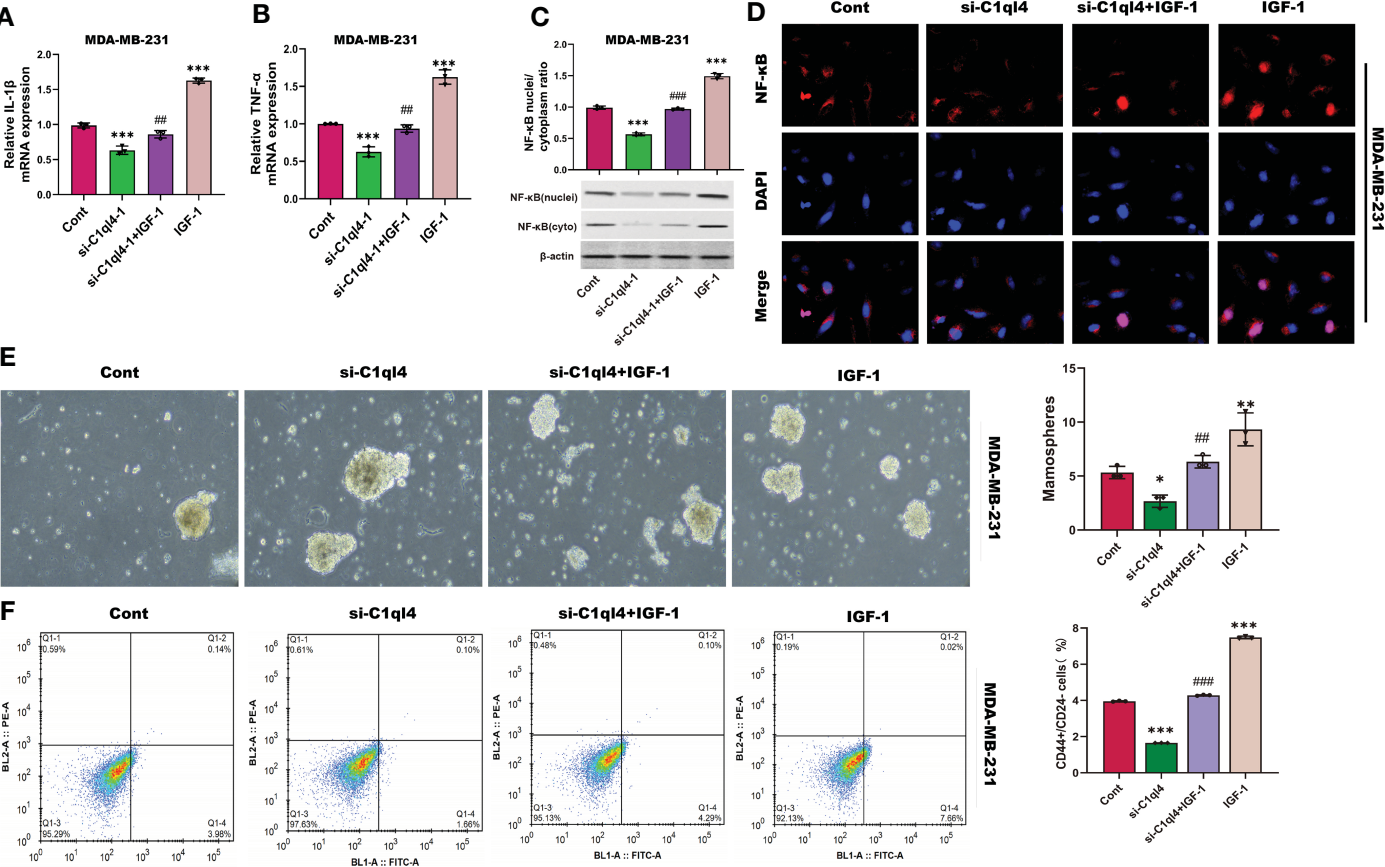
C1ql4 regulates the stemness *via* PI3K/AKT/NF-κB pathway. **(A, B)** The effect of IGF-1 on TNF-α and IL-1β in C1ql4 knockdown MDA-MB-231 cells was detected by qPCR experiment. **(C)** The effect of IGF-1 on NF-κB in C1ql4 knockdown MDA-MB-231 cells was detected by Western blot assay. **(D)** The effect of IGF-1 on NF-κB location in C1ql4-knockdown MDA-MB-231 cells was detected by immunofluorescence assay. **(E)** Sphere formation experiment was used to detect the effect of IGF-1 on sphere formation ability. **(F)** Flow cytometry was employed to verify the effect of IGF-1 on stemness. *p <0.5, **p < 0.01, ***p < 0.001 vs Cont;^##^ *P*<0.01,^###^ *P*<0.001 vs si-C1ql4.

**Figure 9:**
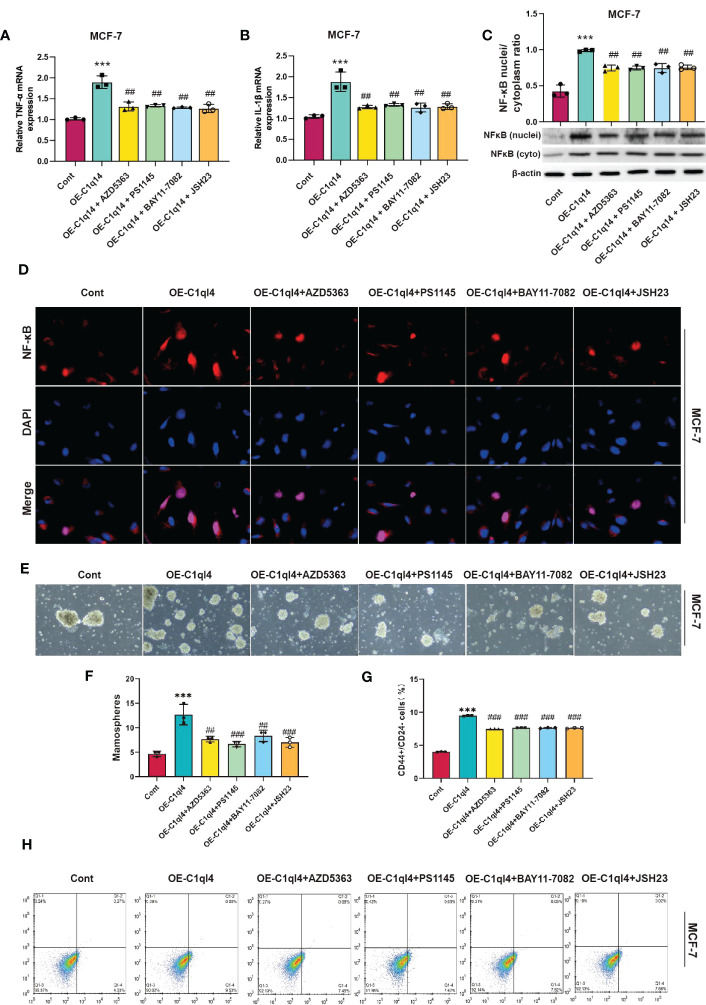
C1ql4 regulates the stemness via PI3K/AKT/NF-kB pathway. **(A, B)** The effect of PI3K/AKT/NF-kB inhibitors on TNF-a and IL-1b in C1ql4 overexpression MCF-7 cells was detected by qPCR experiment. **(C)** The effect of PI3K/AKT/NF-kB inhibitors on NF-kB in C1ql4 overexpression MCF-7 was detected by Western blot assay. **(D)** The effect of PI3K/AKT/NF-kB inhibitors on NF-kB location in C1ql4 overexpression MCF-7 was detected by immunofluorescence assay. **(E, F)** Sphere formation experiment was used to detect the effect of PI3K/AKT/NF-kB inhibitors on sphere formation ability. **(G, H)** Flow cytometry was employed to verify the effect of PI3K/AKT/NF-kB inhibitors on stemness. ***P< 0.001 vs Cont; ##*P*<0.01,###*P*<0.001 vs OE-C1ql4.

Next, we detected whether activation of PI3K/AKT pathway was able to antagonize the inhibitory effects of C1ql4 knockdown on cancer stemness. Consequently, C1ql4-knockdown BC cells were treated with or without IGF-1. We showed that IGF-1 increased the expression of PI3K/AKT target genes (TNF-α, IL-1β) and enhanced nuclear location of NF-κB in C1ql4-lost cells (**Figures 8A–D)**. Furthermore, we demonstrated that activation of AKT diminished the C1ql4 knockdown-mediated suppressive effects on cell stemness (**Figures 8E, F**). At the same time, we explored whether inhibition of AKT pathway could reverse the facilitating effect of C1ql4 overexpression in MCF-7 cells. We found that AKT pathway inhibitor could inhibited the effect of C1ql4 overexpression on the expression of PI3K/AKT target genes (TNF-α, IL-1β, NF-κB) (**Figures 9A–D)** and the formation ability of spheroids and the portion of CD44+CD24-/low cells in MCF-7 cells (**Figures 9E, F**). These data suggested that C1q14 facilitated the cancer stemness in BC cells *via* the PI3K/AKT/NF-κB signaling pathway.

### C1q14 regulates BC cell stemness and EMT *in vivo*

**Figure 10:**
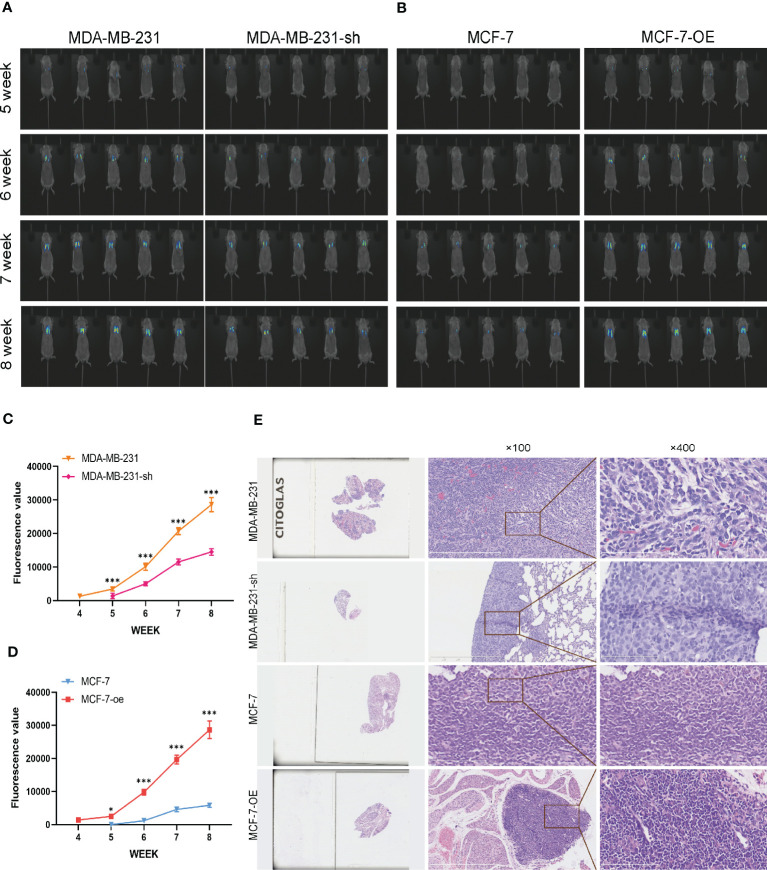
Analysis of BC cells metastasis in mouse lung cells. The MCF-7-luc cells that infected with C1q14 overexpression vectors (MCF-7-oe) and control cells, MDA-MB-231-luc cells that depleted of C1q14 (MDA-MB-231-sh) and control MDA-MB-231 cells were injected through tail vein. **(A)** The fluorescence of MDA-MB-231 and MCF-7 cells metastasis in lung tissues was observed using IVIS at 5, 6, 7 and 8 weeks after injection. **(B, C)** The quantified fluorescence curve of **(C)** MDA-MB-231 and **(D)** MCF-7 cells metastasis. **(D)** The HE staining of lung tissues (magnification: left, 10x; middle, 100x; right, 400x). *P<0.05, ***P<0.001 vs Cont.

**Figure 11:**
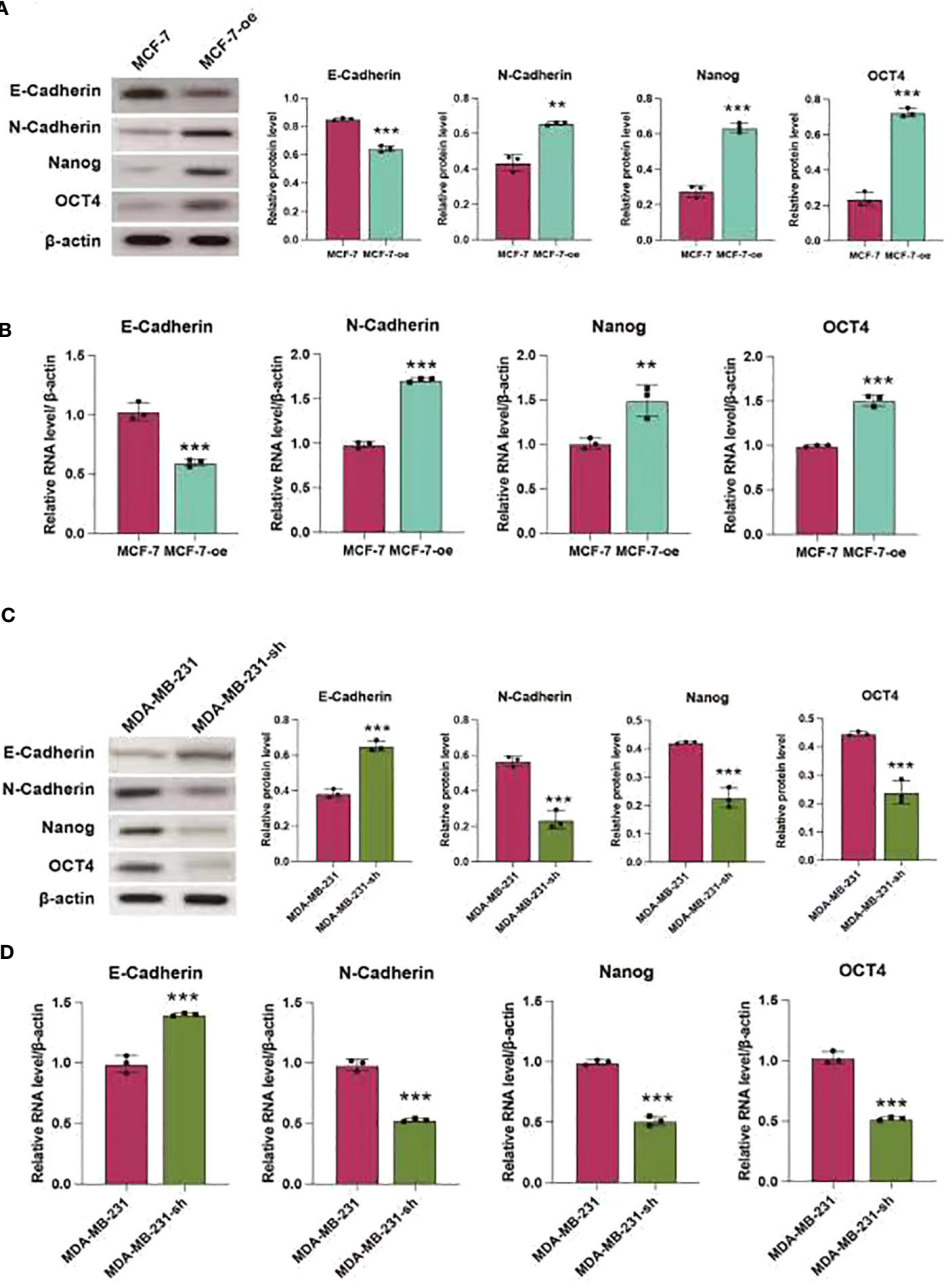
C1q14 regulates stemness and EMT of BC cell in vivo. The MCF-7-luc cells that infected with C1q14 overexpression vectors (MCF-7-oe) and control cells, MDA-MB-231-luc cells that depleted of C1q14 (MDA-MB-231-sh) and control MDA-MB-231 cells were injected through tail vein. **(A, B)** The **(A)** protein and **(B)** RNA levels of epithelial biomarker E-cadherin were decreased and the mesenchymal biomarkers, N-cadherin, Nanog, and OCT4 in MCF-7 tumors were checked by western blotting and qPCR assay. **(C, D)** The (H) protein and (I-N) RNA levels of epithelial biomarker E-cadherin were decreased and the mesenchymal biomarkers, N-cadherin, Nanog, and OCT4 in MDA-MB-231 tumors were checked by western blotting and qPCR assay. **p < 0.01, ***p < 0.001

We further validated the regulatory effects of C1q14 on stemness and EMT using *in vivo* mouse model. We constructed lentivirus vectors for overexpression or knockdown of C1q14 in luciferase-labeled MCF-7 or MDA-MB-231 cells, respectively. Five weeks after tail vein injection of the cells, we observed notable fluorescence in lungs of tumors. Noteworthy, the fluorescence density in lungs of C1q14-overexpressed MCF-7 tumors were significantly stronger than that of control MCF-7 cells (**Figure 10A**). In contrast, the knockdown of C1q14 in MDA-MB-231 cells suppressed the cell metastasis to lung tissues, compared with the control MDA-MB-231 cells (**Figure 10A**). The quantification of fluorescence in metastasis sites showed time-dependent decrease in MDA-MB-231 cells after knockdown of C1q14 (**Figure 10B**) and an increase in C1q14-overexpressed MCF-7 cells (**Figure 10C**). Moreover, the results from HE staining presented increased lung damage and metastasis foci of C1q14-overexpressed MCF-7 cells compared with MCF-7 cells (**Figure 10D**). The C1q14-depleted MDA-MB-231 cells showed decreased lung tissue damages compared with control MDA-MB-231 cells (**Figure 10D**). We then collected the lung tissues for further detection of stemness biomarkers and EMT biomarkers. As shown in **Figures 11A–G**, the protein and RNA levels of epithelial biomarker E-cadherin were decreased and the mesenchymal biomarkers, N-cadherin, Nanog, and OCT4, were elevated in C1q14 overexpressed MCF-7 tumors compared with control MCF-7 tumors. In contrast, the knockdown of C1q14 in MDA-MB-231 tumors significantly upregulated the protein and RNA level of E-cadherin and downregulated N-cadherin, Nanog, and OCT4 level (**Figures 11H–N)**, compared with the control tumors.

## Discussion

Cancer stem cells exhibit the properties of multidirectional differentiation, self-renewal, and high proliferation, which various studies have indicated to participate in initiation, progression, and metastasis of cancers (22). In present study, we demonstrated the role of C1ql4 in promoting stemness and EMT in BC cells. We found that high expression of C1q14 was remarkably correlated with poor prognosis of BC patients. Our data also showed that C1ql4 is highly expressed in CD44+/CD24-/low cells that present BCSCs. Functional studies have found that C1ql4 could positively regulate the self-renewal ability and the portions of CD44+CD24-/low cells in BC cells. Besides, C1ql4 also caused high expression of EMT biomarkers.

Studies have reported that cancer stem cells commonly exhibit EMT features and is involved in cancer metastasis (22). The cancer metastasis is correlated to CSCs that migrate to distant organs and further form new foci (23). Accumulating evidence has proved that cancer cells at the early stage of metastasis exhibit gene expression patterns similar to normal stem cells (23). Numerous signaling pathways are correlated with EMT, such as transforming growth factor beta (TGF-β) signaling pathway, NF-κB pathway, and receptor tyrosine kinase (RTK) signaling pathway, etc. (10, 24) The EMT biomarkers mainly include increased level of N-cadherin, Snail, Nanog, Slug, Vimentin et al, which are transcriptionally or post-transcriptionally regulated by various factors (25, 26). For example, NF-κB and IKK have been reported to interact with the promoter of Snail to upregulate its expression (19). Activated Snail upregulates the expression of other transcription factors related to EMT such as ZEB1/2, further promoting EMT (27). Here, we found that C1ql4 overexpression and knockdown modulated the activation and suppression of NF-κB signaling, manifested by decreased nuclei localization and phosphorylation. The suppressed level of p-NF-κB in nuclei directly impeded its role in promoting gene transcription (28). The altered RNA levels of TNF-α and IL-1β, the two representative target genes of NF-κB, confirmed the role of C1ql4 in modulating NF-κB signaling pathway.

The PI3K/AKT signaling is a critical pathway that participates in a great number of pathological and physiological conditions, including cell proliferation, migration, invasion, differentiation, metabolism, and angiogenesis (29). Activation of the PI3K/AKT pathway in BCSCs is required for the *in vitro* proliferation ability and *in vivo* tumorigenicity (30). The PI3K/AKT pathway also directly regulates the expression of CSC biomarkers, such as the ALDH1, CD133, in colorectal cancer (10, 31). Our further study on regulatory mechanisms indicated that inactivation of PI3K/AKT signaling pathway suppressed the effects of C1ql4 on BC stemness and EMT and the activation of NF-κB signaling. Hence, we identified that C1ql4 modulated PI3K/AKT/NF-κB axis to participate in the metastasis of BC cells.

## Conclusions

We identified that C1ql4 is correlated with elevated BCSC properties, EMT, and metastasis. The molecular study demonstrated that C1ql4 upregulated the PI3K/AKT signaling to activate the function of NF-κB in BCSCs. Our work presented C1ql4 as a potential target for the treatment of metastatic BC.

## Data availability statement

The original contributions presented in the study are included in the article/supplementary material. Further inquiries can be directed to the corresponding authors.

## Ethics statement

The studies involving human participants were reviewed and approved by Medical Ethics Committee of Affiliated Hospital of Chengde Medical University. The patients/participants provided their written informed consent to participate in this study. The animal study was reviewed and approved by Medical Ethics Committee of Affiliated Hospital of Chengde Medical University.

## Author contributions

This study was conceived, designed, and interpreted by FX, QL and LL. JW analyzed the data. Material preparation and data collection were performed by SZ and YD. LL directed the experiments. FX wrote the manuscript. All authors have read and agreed to the published version of the manuscript.
